# Supplementary material for: HER2 Overexpression and Cytogenetical Patterns in Canine Mammary Carcinomas
Source: Vet Sci. 2022 Oct 22;9(11):583. doi: 10.3390/vetsci9110583 (PMC9694975; doi:10.3390/vetsci9110583)

Table S1. FISH method details.

Permeation	Digestion	Probes	Denaturation	Hybridization	Stringency
Citrate buffer pH 8.00 in microwave at 750W for 5 min 30 sec	HCl pepsine at 37°C for 19 min and 30 sec.	*HER2* (red), *CRYBA1* (green) (Empire Genomic LLC, Buffalo, NY).	83°C for 3 min	37°C overnight	NP40 1.5%/2×SSC (pH 7.0–7.5) at 65°C for 50 sec

Table S2: HER2, ER, KI67 data in canine mammary carcinoma.

ID	Histological grade	Histological diagnosis	HER2 IHC	*HER2* gene copies	*CRYBA1* gene copies	Fusion	Cluster-type amplification	co-amplification	*HER2*/*CRYBA1* ratio	Amplification status (single probe)	Amplification status dual probe	Ki67 index	ER score
AP1749-1	2	complex carcinoma	1	nd	nd	0	0	0	nd	nd	nd	20.16%	3
AP2143-2	3	comedocarcinoma	2	3.97	2.47	0	0	0	1.6	0	0	36.65%	4
AP3272 V1-1	1	mixed carcinoma	1	nd	nd	nd	nd	nd	nd	nd	nd	14.82%	4
AP3547 3A-4A	2	anaplastic carcinoma	0	nd	nd	0	0	0	nd	nd	nd	29.96%	0
AP3676-1	1	complex carcinoma	2	nd	nd	0	0	0	nd	nd	ND	8.26%	3
AP3728-2	2	complex carcinoma	2	nd	nd	0	0	0	nd	nd	ND	nd	nd
AP3935 1A	1	complex carcinoma	1	5.12	2.96	0	0	0	1.73	0	0	5.87%	0
AP3935 2A	2	complex carcinoma	3	6.06	3.75	1	1	0	1.61	1	1	12.45%	8
AP3951-1	1	mixed carcinoma	2	nd	nd	0	0	0	nd	nd	nd	15.78%	3
AP4585 V1-1	2	infiltrative micropapillary carcinoma	2	4.42	5.6	0	0	1	0.83	0	0	33.28%	0
AP4699	1	lipid rich carcinoma	2	nd	nd	0	0	0	nd	nd	nd	9.27%	4
AP4906	3	lipid rich carcinoma	0	1.68	1.75	0	0	0	0.96	0	0	60.93%	0
AP4965	2	mixed carcinoma	3	1.95	1.95	0	0	0	1	0	0	38.89%	0
AP5604	1	simple tubulo-papillary carcinoma	1	nd	nd	nd	nd	nd	nd	nd	nd	32.18%	0

AP6075-1	1	complex carcinoma	1	1.93	1.88	0	0	0	1.03	0	0	nd	nd
AP6162-1	3	inflammatory carcinoma	1	2.35	1.95	0	0	0	1.2	0	0	57.49%	0
AP6187	3	simple solid carcinoma	2	1.4	1.81	0	0	0	0.77	0	0	10.60%	0
AP6794	2	complex carcinoma	0	nd	nd	0	0	0	nd	nd	nd	nd	nd
AP6825	1	mixed carcinoma	0	1.93	1.81	0	0	0	1.07	0	0	9.82%	0
AP6996	1	complex carcinoma	1	1.55	1.73	0	0	0	0.89	0	0	18.53%	3
AP7011	1	mixed carcinoma	1	1.62	1.77	1	0	1	0.91	0	0	6.86%	0
AP7122	1	simple tubulo-papillary carcinoma	1	1.62	1.77	1	0	0	0.91	0	0	3.02%	0
AP7308-7	2	simple solid carcinoma	0	4.43	2.57	0	0	0	1.72	0	0	38.33%	0
AP7359	1	mixed carcinoma	3	2.06	1.93	1	0	0	1.06	0	0	30.49%	5
AP7530	3	adenosquamous carcinoma	1	2.23	1.21	0	0	0	1.84	0	0	17.09%	3
AP7559	1	simple tubular carcinoma	3	2.05	2	0	0	0	1.025	0	0	18.83%	5
AP7628	1	simple tubulo-papillary carcinoma	2	1.68	1.8	0	0	0	0.94	0	0	nd	nd
AP7789	1	mixed carcinoma	3	2,6	2,53	0	0	0	1.082	0	0	4.12%	0
AP7868-1	3	simple solid carcinoma	3	1.71	1.94	1	0	0	0.88	0	0	63.80%	0
AP7868-2	3	simple tubulo-papillary carcinoma	3	1,85	1,69	0	0	0	1,09	0	0	5.85%	6
AP7868-3	1	complex carcinoma	2	2	2.29	1	0	0	0.88	0	0	nd	nd
AP7986	1	mixed carcinoma	2	2.81	1.78	1	0	0	1.58	0	0	12.15%	nd

AP8043	1	intraduttal papillary carcinoma	2	1.75	1.78	1	0	0	0.99	0	0	10.23%	0
AP8090	3	comedocarcinoma	3	2.73	2.93	0	0	0	0.93	0	0	26.33%	0
AP8102	2	complex carcinoma	3	5.28	2.03	0	0	0	2.59	1	1	nd	nd
AP8153-2M	1	complex carcinoma	3	4	3.05	0	0	0	1.31	0	0	4.49%	0
AP8153-3M1	1	complex carcinoma	2	1.85	2.08	0	0	0	0.89	0	0	6.29%	0
AP8153-3M2	1	complex carcinoma	2	1.33	1.38	0	0	0	0.96	0	0	7.92%	0
AP8153-4M	1	complex carcinoma	2	2.81	1.9	1	0	0	1.47	0	0	6.14%	0
AP8198	1	complex carcinoma	1	2.54	3.38	0	0	1	0.75	0	0	28.17%	4
AP8643	1	complex carcinoma	3	3.21	2.69	0	0	0	1.19	0	0	10.67%	3
AP8797	2	complex carcinoma	2	2.09	1.72	0	0	0	1.21	0	0	17.62%	3
AP8809	1	complex carcinoma	3	3.7037	nd	0	0	0	nd	0	nd	nd	nd
AP8823	2	simple solid carcinoma	3	3.27	3.2	0	0	1	1.02	0	0	23.84%	7
AP8845	3	simple solid carcinoma	1	1.65	1.74	0	0	0	0.95	0	0	50.92%	0
AP8868	1	mixed carcinoma	2	2.5	2.19	0	0	0	1.14	0	0	8.51%	0
AP8869	3	comedocarcinoma	2	2.7	2.56	0	0	0	1.06	0	0	37.60%	0
AP8875	1	complex carcinoma	3	3.44	2.55	0	0	0	1.34	0	0	18.46%	0
AP8876	1	complex carcinoma	1	7.18	4.95	0	0	1	1.45	1	0	3.90%	6
AP8884	1	complex carcinoma	3	2.18	2.18	0	0	0	1	0	0	14.84%	6
AP8955	1	simple tubular carcinoma	2	1.33	1.54	0	0	0	0.86	0	0	34.16%	6
AP9207	3	simple tubulo-papillary carcinoma	1	15.52	2.91	0	1	0	5.33	1	1	14.31%	0

AP9208	1	mixed carcinoma	2	2.42	2.08	0	0	0	1.17	0	0	8.49%	0
AP9210	1	mixed carcinoma	2	2.29	2.45	1	0	0	0.93	0	0	14.42%	3
AP9393	2	mixed carcinoma	2	4.9	2.33	0	0	0	2.1	1	1	21.70%	0
AP9398-1	1	complex carcinoma	2	2.27	3.16	0	0	0	0.72	0	0	24.04%	0
AP9398-5	2	simple tubulo-papillary carcinoma	1	5.4	4.88	0	0	1	1.1	0	0	29.50%	0
AP9485	1	complex carcinoma	2	2.67	2.5	0	0	0	1.06	0	0	21.72%	0
AP9581	3	comedocarcinoma	1	nd	nd	nd	nd	nd	nd	nd	nd	53.69%	0
AP9596-1	2	intraduttal papillary carcinoma	2	1.91	1.74	0	0	0	1.1	0	0	29.19%	0
146/14	1	simple tubular carcinoma	1	2.45	2.45	0	0	0	1	0	0	57.00%	0
221/14 4-1	3	simple solid carcinoma	2	1.8	1.27	1	0	0	1.41	0	0	17	nd
221/14 4-2	3	comedocarcinoma	2	2	2	1	0	0	1	0	0	14	0
518/14	1	mixed carcinoma	0	1.44	1.6	0	0	0	0.9	0	0	12	4
018/15	1	simple tubulo-papillary carcinoma	0	nd	nd	0	0	0	nd	nd	nd	5	0
030/15 D	3	simple solid carcinoma	2	5.54	2.02	0	0	0	2.73	1	1	49	0
054/15 A	1	complex carcinoma	1	nd	nd	0	0	0	nd	nd	nd	7	3
057/15	2	complex carcinoma	2	nd	nd	0	0	0	nd	nd	ND	1	3
058/15	3	simple solid carcinoma	1	nd	nd	nd	nd	nd	nd	nd	nd	17	nd
059/15 B	~~1~~	complex carcinoma	2	2.66	2.04	0	0	0	1.32	0	0	4	3
088/15 3-4	2	duttal carcinoma	1	3.73	1.66	1	1	0	2.24	0	0	3	

100/15	1	simple tubulo-papillary carcinoma	0	1.76	2.17	1	0	0	0.81	0	0	6	6
111/15 _sx	1	complex carcinoma	1	1.66	2.28	0	0	0	0.73	0	0	11	
160/15 1	2	mixed carcinoma	2	nd	nd	0	0	0	nd	nd	nd	nd	3
160/15 2	1	simple tubulo-papillary carcinoma	0	1.75	1.33	0	0	0	0.76	0	0	7	0
160/15 4	1	simple tubular carcinoma	1	nd	nd	0	0	0	nd	nd	nd	5	3
181/15 3	3	micropapillary carcinoma	1	1.125	1.63	0	0	0	0.69	0	0	31	nd
189/15 SX	1	complex carcinoma	2	1.79	1.65	0	0	0	1.08	0	0	20	3
190/15	3	simple solid carcinoma	1	1.69	2.07	0	0	0	0.82	0	0	17	nd
194/15	1	simple tubulo-papillary carcinoma	1	1.75	2.08	0	0	0	0.84	0	0	17	4
201/15	1	mixed carcinoma	1	2.5	2.11	0	0	0	1.18	0	0	5	0
224/15 -1	2	complex carcinoma	1	1.43	1.87	0	0	0	0.76	0	0	16	nd
224/15 -2	2	complex carcinoma	2	2.34	2.25	0	0	0	1.04	0	0	17	5
281/15 -1	1	simple tubulo-papillary carcinoma	0	6.15	2.75	0	1	0	2.24	1	1	nd	4
281/15 -2	1	intraduttal papillary carcinoma	1	2.37	2.55	0	0	0	0.93	0	0	4	6
281/15 -3	1	simple tubulo-papillary carcinoma	0	2.47	1.45	0	0	0	1.7	0	0	1	4
282/15 -1	2	mixed carcinoma	1	3.92	2.25	0	0	0	1.74	0	0	nd	3

282/15-2	1	simple tubular carcinoma	1	nd	nd	0	0	0	nd	nd	nd	7	5
282/15-3	3	complex carcinoma	2	1.38	1.55	0	0	0	0.88	0	0	32	0
298/15	1	mixed carcinoma	1	1.89	1.84	0	0	0	1.03	0	0	14	3
359/15	1	mixed carcinoma	2	2.36	2.17	1	0	0	1.08	0	0	4	0
400/15	2	complex carcinoma	1	nd	nd	nd	nd	0	nd	nd	nd	16	3
401/15	3	simple solid carcinoma	1	1.88	2.65	1	0	0	0.71	0	0	nd	0
475/15	3	simple solid carcinoma	1	1.06	2.33	0	0	0	0.46	0	0	33	0
489/15	2	inflammatory carcinoma	1	1.88	1.72	1	0	0	1.09	0	0	38	0
518/15-1	3	simple solid carcinoma	0	1.93	2.44	0	0	0	0.8	0	0	40	0
518/15-2	2	simple solid carcinoma	0	1.75	1.8	0	0	0	0.97	0	0	34	0
537/15	2	complex carcinoma	0	nd	nd	0	0	0	nd	nd	ND	21	0
565/15	1	complex carcinoma	2	2.65	1.77	1	0	0	1.49	0	0	nd	4
576/15	1	simple tubulo-papillary carcinoma	1	nd	nd	0	0	0	nd	nd	nd	4	3
584/15-1	1	complex carcinoma	1	1.85	1.63	1	1	0	1.13	0	0	nd	nd
584/15-2	1	complex carcinoma	1	1.63	1.75	0	0	0	0.93	0	0	nd	nd
602/15	1	simple tubulo-papillary carcinoma	1	4.24	1.72	0	1	0	2.47	0	1	12	6
614/15	3	simple solid carcinoma	1	1.85	2.85	0	0	0	0.65	0	0	36	3
618/15-A	3	simple solid carcinoma	1	3.57	4.91	0	0	1	0.73	0	0	38	0

618/15 -B	3	simple solid carcinoma with neuroendocrine differentiation	2	2.19	2.73	0	0	0	0.8	0	0	55	0
618/15 -C2	3	comedocarcinoma	1	2.15	2.09	0	0	0	1.03	0	0	nd	0
618/15 -D1	3	simple solid carcinoma	1	1.47	1.91	0	0	0	0.77	0	0	nd	nd
001/16	2	simple tubulo-papillary carcinoma	2	4.38	1.97	0	0	0	2.22	1	1	10	nd
087/16	1	simple tubular carcinoma	0	1.72	1.9	1	0	0	0.91	0	0	7	nd
101/16	1	mixed carcinoma	1	1.8	1.87	1	0	0	0.96	0	0	4	3

Figure S1. Original blots.

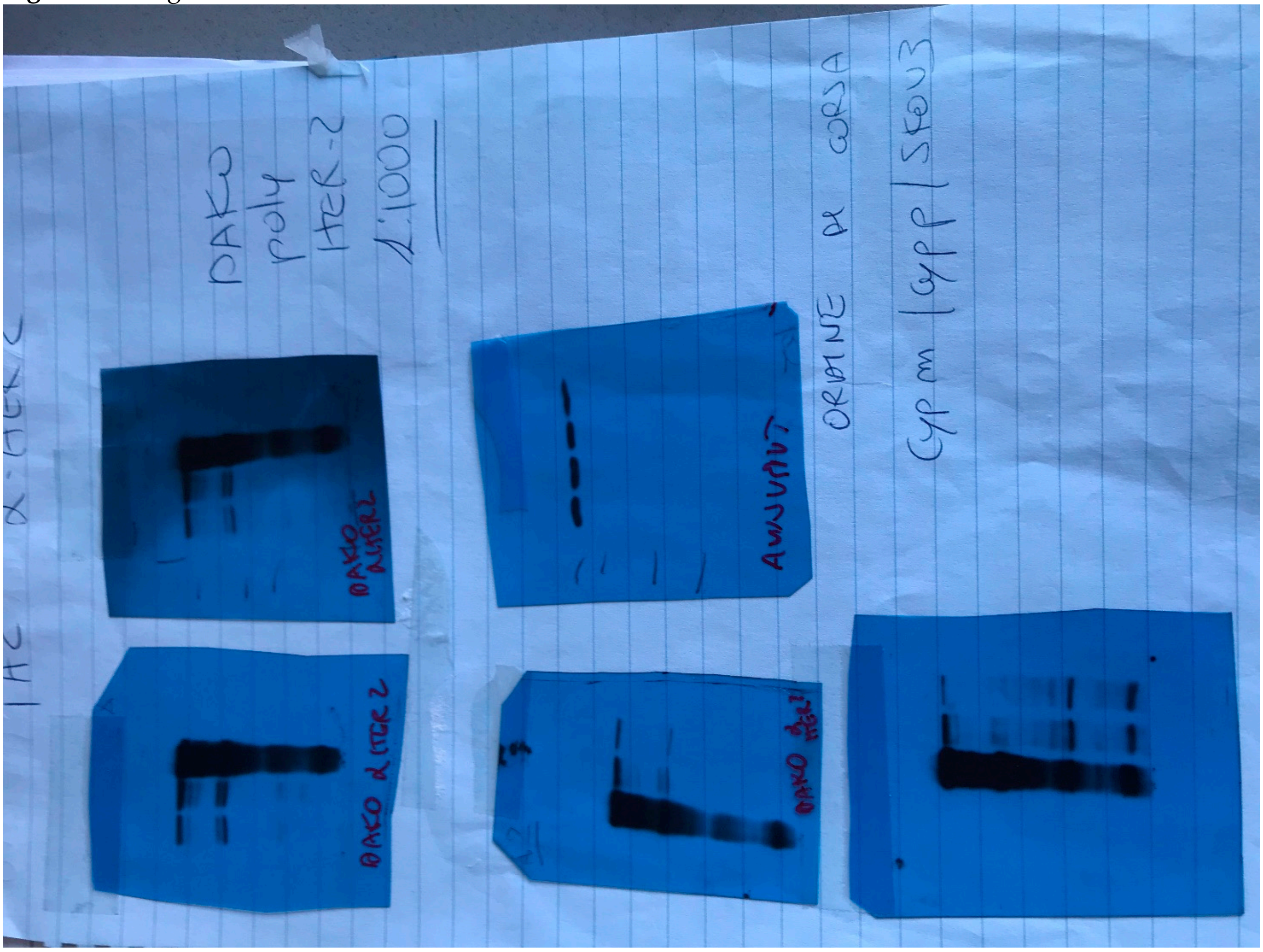

Supplement: Supplementary file 1 [file vetsci-09-00583-s001.zip › vetsci-1908319-supplementary.pdf]
